# Phylogenetic and Structural Analysis of Miconazole Susceptibility in *Malassezia pachydermatis* Isolates From Dogs With Otitis Externa

**DOI:** 10.1111/vde.70059

**Published:** 2026-03-19

**Authors:** Cole M. Belcher, Clarissa P. Souza, Elyas McGuire, Chien‐Che Hung, Joel D. A. Tyndall, Lois L. Hoyer

**Affiliations:** ^1^ Department of Pathobiology University of Illinois College of Veterinary Medicine Urbana Illinois USA; ^2^ Department of Veterinary Clinical Medicine University of Illinois College of Veterinary Medicine Urbana Illinois USA; ^3^ School of Pharmacy University of Otago Dunedin New Zealand; ^4^ Veterinary Diagnostic Laboratory University of Illinois Urbana‐Champaign Urbana Illinois USA

**Keywords:** CYP51, *ERG11*, *Malassezia pachydermatis*, miconazole, otitis externa, phylogeny

## Abstract

**Background:**

An increased number of dogs with unresolved yeast otitis externa (OE) after miconazole treatment was observed at a tertiary practice.

**Hypothesis/Objectives:**

To evaluate miconazole susceptibility in *Malassezia pachydermatis* isolates from canine OE.

**Animals:**

Eleven client‐owned dogs (16 ears) with clinical and video otoscopic signs of OE and cytological evidence of yeast.

**Materials and Methods:**

Yeast cultures were derived from ear swabs. PCR amplified portions of the internal transcribed spacer region (ITS), and partial sequences encoding the 18S rRNA small subunit (SSU), 26S rRNA large subunit (LSU), and actin (*ACT1*) loci for phylogenetic analysis, as well as the *ERG11* gene that encodes lanosterol 14α‐demethylase. Miconazole susceptibility was assessed by disk diffusion on Mueller–Hinton agar. Molecular modelling was used to evaluate the relationship between lanosterol 14α‐demethylase amino acid substitutions and miconazole susceptibility.

**Results:**

Isolates of *M. pachydermatis* from canine OE formed a three‐clade phylogeny. Most *ERG11* mutations predicted lanosterol 14α‐demethylase substitutions that were clade‐specific without an effect on miconazole susceptibility. Reduced susceptibility was associated with substitutions of amino acid A302, located in the lanosterol 14α‐demethylase azole‐binding site. Other altered amino acids were located elsewhere in the lanosterol 14α‐demethylase protein without an apparent effect on miconazole binding.

**Conclusions and Clinical Relevance:**

Clinical isolates of *M. pachydermatis* with reduced miconazole susceptibility were easily found, suggesting that miconazole resistance is present in routine patients. Lanosterol 14α‐demethylase interactions with longer‐tailed azoles involve additional contacts beyond A302 suggesting these drugs will be more effective against miconazole‐resistant strains. However, effective antifungal stewardship is needed to slow development of pan‐azole resistance that would limit treatment options for dogs with *M. pachydermatis*‐associated disease.

## Graphical abstract

Background: An increased number of dogs with unresolved yeast otitis externa (OE) after miconazole treatment was observed at a tertiary practice. Hypothesis/Objectives: To evaluate miconazole susceptibility in *Malassezia pachydermatis* isolates from canine OE. Conclusions and Clinical Relevance: Reduced susceptibility to miconazole was associated with substitution of amino acid A302 in the Erg11 drug target. Other Erg11 amino acid substitutions largely reflected phylogenetic differences among *M. pachydermatis* isolates rather than reduced miconazole susceptibility. Lanosterol 14α‐demethylase interactions with longer‐tailed azoles involve additional contacts beyond A302 suggesting these drugs will be effective against miconazole‐resistant strains. However, effective antifungal stewardship is needed to slow the development of pan‐azole resistance that would limit treatment options for dogs with *M. pachydermatis*‐associated disease.

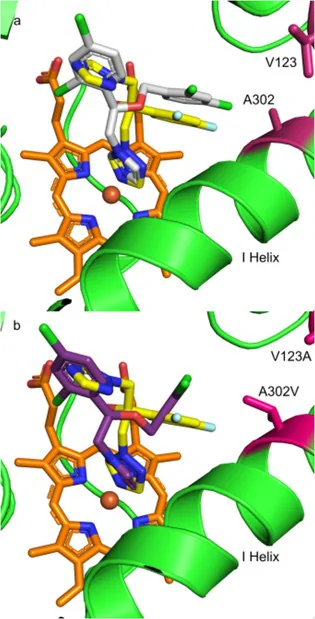

## 1 Introduction

Otitis externa (OE) is a common inflammatory condition, estimated to occur in ≤ 20% of dogs seen by veterinary surgeons worldwide [1, 2]. An underlying allergic disease often predisposes dogs to OE and is frequently complicated by secondary bacterial and/or *Malassezia pachydermatis* infections.

Canine *Malassezia* OE is typically treated with topical and/or systemic azole antifungal medications, potentially combined with glucocorticoids to address the underlying disease and provide comfort. Persistent or recurrent cases of *Malassezia* OE reportedly are increasing [3, 4]. Experience at a referral dermatology and otology service supported these findings and suggested the potential emergence of miconazole resistance.

The objective of this study was to evaluate miconazole susceptibility in *M. pachydermatis* isolates from dogs with OE. Phylogenetic analysis was used to assess the evolutionary relationships within the collection of isolates. This information was then used to assess the potential impact of amino acid substitutions in lanosterol 14α‐demethylase (encoded by the *ERG11* gene) on miconazole susceptibility.

## 2 Materials and Methods

### 2.1 Ethics

This study was approved by the Institutional Animal Care and Use Committee (protocol 23046). Informed owner consent was obtained before enrollment of each dog.

### 2.2 Animals

Dogs of any age, breed, or sex with clinical and video otoscopic diagnosis of OE and the presence of yeast on cytological evaluation were eligible for the study. The 0–3 Otitis Index Score (OTIS3) for erythema, oedema/swelling, erosion/ulceration and exudate was used to characterise the OE clinical severity in dogs upon enrollment. Eleven dogs (16 ears) were enrolled in the study, and each enrolled ear had a total OTIS3 ≥ 4 (Table 1) [5].

**TABLE 1:** Dog signalment and 0–3 Otitis Index Score (OTIS3) for each sampled ear.

Dog	Sex	Age at enrollment	Breed	Ear(s) sampled	Total OTIS3
1	FS	4 years	Pit bull mix	Left	5
2	FS	6 years 4 months	Labrador retriever	Right	4
4	MN	7 years 9 months	French bulldog	Left	7
5	FS	11 years 5 months	Standard poodle	Right	5
				Left	5
6	FS	1 year 11 months	Goldendoodle	Right	7
				Left	8
7	MN	4 years 10 months	Labradoodle	Right	4
8	FS	8 years 5 months	Labrador retriever	Right	4
				Left	5
9	MN	1 year 9 months	Labrador retriever mix	Right	9
10	MN	3 years 10 months	Cocker spaniel	Left	5
11	MN	9 years 3 months	Bassett hound	Right	10
				Left	10
12	FS	3 years 8 months	Pit bull	Right	10
				Left	11

Abbreviations: FS, female spayed; MN, male neutered.

### 2.3 Ear Sampling and Yeast Isolates

A sterile swab was used to sample each affected ear individually. The swabs were submitted to the Veterinary Diagnostic Laboratory for microbiological culture on Sabouraud dextrose agar (SDA) and potato dextrose agar, both with and without chloramphenicol. Dry‐to‐waxy cream‐coloured colonies were screened microscopically for an ellipsoidal cell morphology characteristic of *M. pachydermatis*. Pure cultures were derived, suspended in tryptic soy broth (TSB; Remel catalogue no. R455052) with 50% glycerol, and stored at −80°C. The *M. pachydermatis* type strain, ATCC 14522 (CBS 1879), was purchased from the American Type Culture Collection (ATCC).

### 2.4 DNA Sequencing

Genomic DNA was used as the template for PCR amplification of *M. pachydermatis* loci. To isolate genomic DNA, colonies were scraped from a SDA plate and suspended in 10 mm Tris‐hydrochloric acid (HCl)/1 mm ethylenediaminetetraacetic acid (EDTA) (TE buffer), pH 8.0. Glass beads (425–600 μm, sterile) and an equal volume of phenol: chloroform (1:1) were added to the tube which was then vortexed to break the cells. DNA was collected from the aqueous phase by isopropanol precipitation then treated with RNAse A, phenol: chloroform‐extracted and isopropanol‐precipitated. DNA was dissolved in 50 μL TE pH 8.0 and stored at −20°C.

Four genetic loci were included in the phylogenetic analysis: the internal transcribed spacer region (ITS), and partial sequences encoding the 18S rRNA small subunit (SSU), 26S rRNA large subunit (LSU) and actin (*ACT1*). PCR primers used to amplify and sequence each locus are detailed in Table S1. PCR used Q5 High‐Fidelity DNA Polymerase (New England BioLabs), a proofreading polymerase to promote the accuracy of the resulting amplicons. Fifty‐microlitre reactions were assembled according to the manufacturer's instructions without including the High GC Enhancer. Reactions were heated at 98°C for 1 min, followed by 35 cycles of 98°C for 10 s/60°C for 30 s/72°C for 30 s, and a final 2 min at 72°C. The annealing temperature for the ITS primers was 52°C instead of 60°C.

The DNA sequence of the *ERG11* gene was derived for each yeast isolate. PCR primers are shown in Table S2. PCR used Phusion, a high‐fidelity, proofreading DNA polymerase (Thermo Fisher Scientific). The complete *ERG11* locus with some flanking sequences was amplified using primers MpERG11F and MpERG11R (Table S2) [6, 7]. A 50 μL amplification reaction contained 10 μL of 5× Phusion HF buffer, 4 μL of 2.5 mm dNTPs, 2.5 μL each of 10 mm PCR primer stocks, 1 μL of template genomic DNA, 0.5 μL of Phusion enzyme and 29.5 μL of nuclease‐free water. The PCR program included a denaturation step (98°C for 30 s) followed by 35 cycles of 98°C for 10 s/65°C for 30 s/72°C for 30 s, and a final 7 min at 72°C.

Agarose gel electrophoresis was used to validate a single band and the size of each PCR product. PCR products were purified using the MultiScreen HTS 96‐well filtration system (Millipore) then resuspended in 50 μL sterile MilliQ water at a concentration of approximately 30–50 ng/μL. Sanger sequencing was performed in the campus core DNA Services Laboratory.

*ERG11* DNA sequences were assembled from forward and reverse reads with the aid of EMBL‐EBI sequence analysis tools (https://www.ebi.ac.uk/services) [8] and the Sequence Manipulation Suite (https://www.bioinformatics.org/sms2) [9]. Each nucleotide was validated by at least two independent reads. DNA sequences were deposited into GenBank (Table S3). *ERG11* DNA sequences were translated into amino acids using the Expasy Translate Tool (https://web.expasy.org/translate) and the standard genetic code.

### 2.5 Phylogenetic Analysis

For the ITS, SSU, LSU and *ACT1* loci, each reverse primer sequence was passed through the Sequence Manipulation Suite reverse complement tool (https://www.bioinformatics.org/sms2) [9] and the resulting sequence aligned with the forward primer sequence using clustal omega (https://www.ebi.ac.uk/jdispatcher/msa/clustalo) [8]. The final analysis used only base pairs for which sequence was verified using both the forward and reverse reactions. For sequence positions that did not match between the forward and reverse reads, trace files were opened in snapgene viewer v8.0.3 (https://www.snapgene.com). The nucleotide with the higher quality score was kept. All sequences for each amplicon were trimmed to the same starting and ending base pair manually. GenBank accession numbers for the DNA sequences are listed in Table S3.

*Malassezia caprae* CBS 10434 was included in the phylogenetic analysis as the outgroup. For each locus, Fasta files were aligned with Mafft v7.310 using the auto command [10]. For each gene, the L‐INS‐i option was auto chosen. modeltest‐ng v0.1.7 was used to choose the most suitable DNA evolutionary substitution model for each Fasta file separately [11]. The TrN, TrN+I, K80+G4 and HKY+G4 models were chosen for SSU, LSU, ITS and *ACT1*, respectively. Individual Fasta files were concatenated manually in the order of SSU‐LSU‐ITS‐*ACT1* for a total of 3050 characters. IQ‐Tree v2.3.5 was used to generate a maximum‐likelihood (ML) phylogenetic tree, as well as analysing 1000 bootstrap replicates to assess branch support [12]. A partition file was utilised to specify the substitution model for each locus: SSU = 1–782, LSU = 783–1330, ITS = 1331–2053, *ACT1* = 2054–3050. A seed of 26,000 was specified for reproducibility and *M. caprae* stated as the outgroup. The resulting .contree file was visualised using iTOL v7 [13].

### 2.6 Disk Diffusion Assay

The disk diffusion assay described by Pasquetti et al. [14] was adapted for use. Yeast strains were streaked onto SDA plates, then incubated at 35°C for 3 days. BD Difco Dehydrated Urea Broth (Thermo Fisher Scientific catalogue no. DF0272‐17‐2; stored at 4°C) was prepared according to the manufacturer's instructions. One millilitre of Tween 80 (0.1% v/v; BD Difco Tween 80 catalogue no. DF3118‐15‐6; Thermo Fisher Scientific) and 5 mL of Tween 40 (0.5% v/v; Millipore Sigma catalogue no. P1504) were added and the medium was sterilised using a 0.2 μm PES membrane filter (Thermo Fisher Scientific catalogue no. FB12566510). The medium was stored at 4°C. Several well‐isolated, representative colonies for each yeast strain were suspended in the modified Urea Broth.

Because there was considerable clumping of the yeast cells, 0.33 g of autoclaved glass beads (425–600 μm; Millipore Sigma catalogue no. G8772) were added to each tube. Tubes were vortexed at top speed for 2 min to dissociate clumps. Cells were counted using a haemacytometer to quantify the relationship between cell number and spectrophotometer reading at 530 nm (optical density OD_530_). An OD_530_ reading of 0.75 was observed for approximately 2.5 × 10^7^ cells/mL. An OD_530_ reading of 1.0 was selected for preparation of cell suspensions, providing an inoculum toward the upper end of the range of 1 × 10^7^ to 5 × 10^7^ cells/mL that was recommended by Pasquetti et al. [14].

BD Difco dehydrated Mueller–Hinton agar (MHA) medium (Thermo Fisher Scientific catalogue no. DF0252‐17‐6) was prepared according to the manufacturer's instructions. Glucose (20 g/L) and methylene blue (0.5 μg/mL final concentration; diluted from Millipore Sigma catalogue no. 03978) were added before autoclaving [14]. The solution was cooled and 28 mL each distributed by pipette into 100 mm diameter Petri dishes (Thermo Fisher Scientific catalogue no. FB0875712) as recommended in Clinical and Laboratory Standards Institute (CLSI) document M44‐A2 [15]. Plates were cooled and stored at 4°C for ≤ 1 week before use.

A sterile swab was dipped into the *M. pachydermatis* inoculum, then rubbed thoroughly across the surface of a modified MHA plate three times, rotating the plate 90° after each time, to create a lawn of growth. Plates were left upright with the lid slightly ajar inside a biosafety cabinet to air‐dry the inoculum. A miconazole Neo‐Sensitab (10 μg; Rosco Diagnostica, distributed by Key Scientific Products Inc.) was placed onto the centre of each plate using sterile forceps. Plates were inverted and incubated at 37°C. Zones of inhibition were monitored at 24 h intervals. *M*. *pachydermatis* isolates exhibited considerable variation in growth rate with some zones of inhibition maturing at 48 h and others requiring 96 h incubation. Because zone size for the faster‐growing isolates did not change with additional incubation, 96 h was used as the time point for final measurement of zone diameter, which was evaluated with a plastic ruler, rounding to the nearest millimetre. Diameters were measured at right angles; if the zone of inhibition was not circular (i.e., one measurement was ≥ 1 mm narrower than the other), the smaller value was reported. The *M. pachydermatis* type strain ATCC 14522 was used as a standard of comparison for the clinical isolates collected in this study. The assay was repeated twice on separate dates and data reported individually.

### 2.7 Molecular Modelling

Homology models of *M. pachydermatis* lanosterol 14α‐demethylase (CYP51, the product of the *ERG11* gene) were generated with MODELLER 9.22 [16] using the crystal structure of *Saccharomyces cerevisiae* CYP51 in complex with fluconazole (PDB ID: 4WMZ) as a template [17]. Initial models were built incorporating fluconazole as a reference. The heme cofactor generated by MODELLER was replaced with that from the crystal structure (PDB ID: 4WMZ) to facilitate docking studies. Docking of azoles was carried out using GOLD v2025.1.0 flexible ligand docking software [18] with the CYP450 goldscore or chemscore template, the azole moiety from fluconazole as a similarity template, and a distance constraint of 2.05–2.15 Å for the azole–iron (Fe) (heme)‐distance to facilitate correct metal coordination. pymol v3.1.3 (https://www.pymol.org) was used to visualise the modelling results.

## 3 Results

### 3.1 Phylogenetic Analysis of OE Yeast Isolates

**FIGURE 1:**
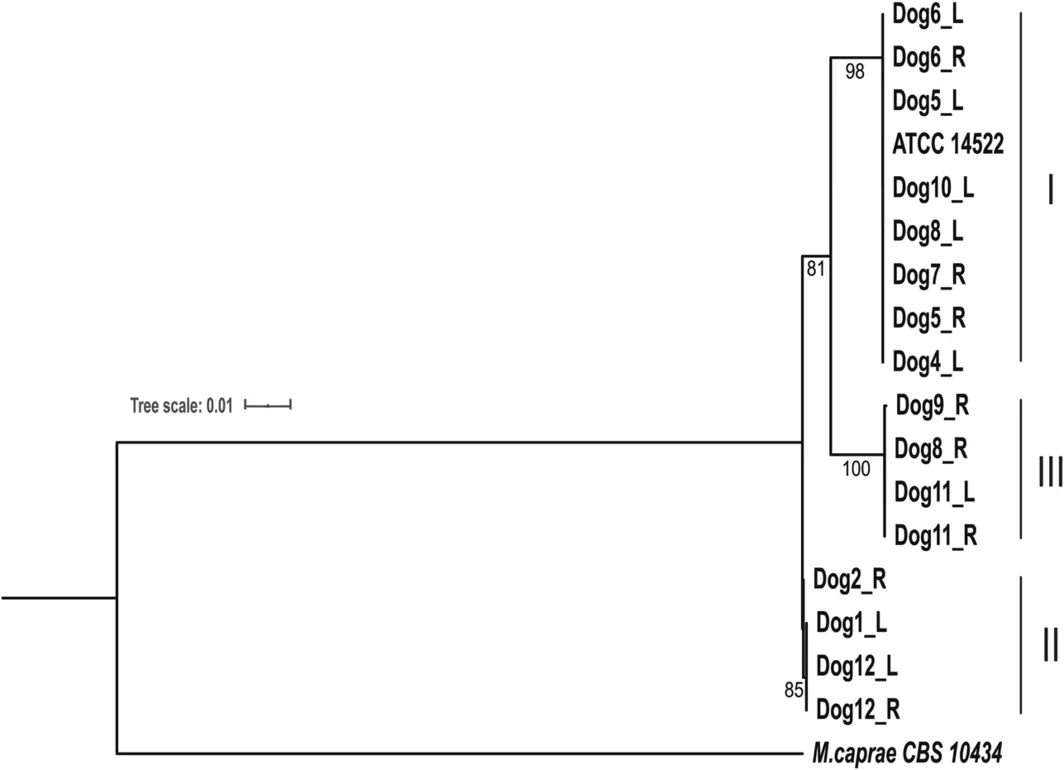
Phylogenetic tree generated using maximum‐likelihood analysis. The tree was derived from the concatenated sequences of the 18S rRNA gene (SSU), 26S rRNA gene (LSU), ITS region and *ACT1* gene. Maximum‐likelihood bootstrap support values ≥ 70% calculated from 1000 replicates are shown on the tree. *Malassezia caprae* CBS 10434 was included as an outgroup. The defined clades (I, II, III) are indicated at the right of the tree. A scale bar representing substitutions per site is shown.

Yeast was recovered into pure culture from 11 dogs. Because five of these dogs had an isolate from both ears, the analysis included a total of 16 yeast specimens which were numbered sequentially. ‘L’ or ‘R’ was appended to indicate isolation from the left or right ear, respectively. The ML phylogenetic tree had a three‐clade evolutionary relationship structure (Figure 1). Clade I was well‐supported with a bootstrap value of 98%; Clade I included the type strain (ATCC 14522) and clinical isolates 4_L, 5_L, 5_R, 6_R, 6_L, 7_R, 8_L and 10_L. Clade II included clinical isolates 1_L, 2_R, 12_L and 12_R, while Clade III contained isolates 8_R, 9_R, 11_L and 11_R. Notably, the isolate from the right ear of Dog 8 was in Clade III, while the isolate from the left ear was in Clade I.

DNA sequences of the ITS region were between 692 and 709 nucleotides in length with 32 informative base pairs (ibp) identified by the IQ‐Tree program. An ibp is defined as a position in the sequence alignment that contains a polymorphism in at least two different isolates (https://iqtree.github.io). The *ACT1* sequence had 997 nucleotides with 45 ibp. The SSU sequence was 782 nucleotides with 2 ibp, while the LSU sequence had 547 nucleotides with 5 ibp.

### 3.2 Assessing Miconazole Susceptibility

A disk diffusion assay was used to evaluate miconazole susceptibility of the clinical isolate collection relative to the *M. pachydermatis* ATCC 14522 type strain. Although there is no standardised method for measuring antifungal resistance for *M. pachydermatis*, various publications have indicated that the type strain has a relatively low minimum inhibitory concentration (MIC) value for miconazole (e.g., 1–3 μg/mL) predicting clinical efficacy for miconazole with this strain [19, 20, 21]. Three of the clinical isolates showed larger zones of inhibition relative to ATCC 14522, indicating that they are likely to be susceptible to miconazole (Table 2). The other isolates had zone diameters less than that for ATCC 14522, with several strains growing up to the miconazole disk (9 mm zone diameter) and clearly not miconazole‐susceptible.

**TABLE 2:** Miconazole disk diffusion assay results for the collection of *Malassezia pachydermatis* canine otitis externa clinical isolates.

Isolate	Clade	Zone diameter (mm)		
		Assay 1	Assay 2	Mean
1_L	II	34	36	35
7_R	I	34	35	34
9_R	III	31	33	32
ATCC 14522	I	29	31	30
6_L	I	29	30	29
8_L	I	28	30	29
8_R	III	29	29	29
2_R	II	24	28	26
6_R	I	24	25	24
12_L	II	22	22	22
12_R	II	20	23	21
10_L	I	13	14	13
5_L	I	11	11	11
5_R	I	11	11	11
11_L	III	9	9	9
11_R	III	9	9	9
4_L	I	9	9	9

One potential mechanism of miconazole resistance is mutation in the *ERG11* gene. *ERG11* encodes lanosterol 14α‐demethylase that is the target of azole antifungal drugs [22]. Lanosterol 14α‐demethylase is a key enzyme in the pathway for synthesis of ergosterol, the main membrane sterol in fungi. Azole antifungal drugs compete with lanosterol for access to the binding site of lanosterol 14α‐demethylase and inhibit the enzyme, thereby reducing the pool of ergosterol required for cell replication. Lanosterol 14α‐demethylase mutations that reduce miconazole binding will lead to resistance. The DNA sequence of *ERG11* from each clinical isolate was derived using Sanger sequencing to reveal nucleotide substitutions that resulted in an altered lanosterol 14α‐demethylase sequence relative to strain ATCC 14522.

Table 3 lists the 16 clinical isolates by clade in order of increasing zone of inhibition size on the disk diffusion assay. Within Clade I, three isolates did not have any amino acid substitutions compared to ATCC 14522. Changing amino acid 302 from Ala to either Thr or Val (A302T or A302V) was associated with decreased miconazole susceptibility. The E181Q mutation was present in the 8_L isolate yet its zone of inhibition size was similar to the type strain, suggesting that E181Q most likely did not reduce miconazole susceptibility.

**TABLE 3:** Amino acid substitutions predicted from *ERG11* DNA sequences for each *Malassezia pachydermatis* canine otitis externa isolate, arranged by clade and associated with disk diffusion assay zone diameters.

	Isolate (mean zone diameter, mm)	Amino acid substitutions
Clade I	4_L (9)	E181Q, **A302T**, G461D
	5_L (11)	R202H, **A302V**
	5_R (11)	R202H, **A302V**
	10_L (13)	E181Q, G271V, **A302V**
	6_R (24)	None
	8_L (29)	E181Q
	6_L (29)	None
	ATCC 14522 (30)	Serves as the reference sequence
	7_R (34)	None
Clade II	12_R (21)	I25S, W52L, R84K, L86F, E181Q, N212S, G271S, E290D, Y352F, H399R, K446R
	12_L (22)	I25S, W52L, R84K, L86F, E181Q, N212S, G271S, E290D, Y352F, H399R, K446R
	2_R (26)	I25S, W52L, R84K, L86F, E181Q, N212S, E290D, Y352F, H399R, K446R
	1_L (35)	I25S, W52L, R84K, L86F, E181Q, N212S, E290D, Y352F, H399R, K446R
Clade III	11_L (9)	A17T, R84K, V123A, R175H, Q178R, E181Q, **A302V**
	11_R (9)	A17T, R84K, V123A, R175H, Q178R, E181Q, **A302V**
	8_R (29)	A17T, R84K, R175H, Q178R, E181Q
	9_R (32)	A17T, R84K, R175H, Q178R, E181Q

*Note:* Bold type was used to highlight A302 substitutions that were the main drivers of reduced miconazole susceptibility. Underlined text highlighted the location of G271S and V123A substitutions that were also discussed in detail in the main text.

Isolates within clades II and III showed many amino acid substitutions compared to ATCC 14522. These substitutions were clade‐specific and reinforced the phylogenetic relationships displayed in Figure 1. Most notably, almost none of the substitutions in Clade II or Clade III isolates affected miconazole susceptibility compared to ATCC 14522. Potential exceptions included the G271S change in both isolates from Dog 12 that was not present in other Clade II strains and the V123A substitution in both Dog 11 isolates that was not present in other Clade III strains. The presence of the A302V substitution continued to be the main driver of reduced miconazole susceptibility.

Isolates of *M. pachydermatis* that showed reduced miconazole susceptibility were all collected from dogs with a history of azole antifungal use according to clinical records and/or information reported by owners. Dog 4 had a history of allergies and OE with miconazole wipes applied to his skin two to three times per week. Dog 5 had previous OE episodes that were treated with miconazole otic solution and flushed regularly with a ketoconazole‐containing product. Dog 10 had a history of environmental allergies and secondary OE and oral ketoconazole treatment. Dog 11 had chronic OE with ketoconazole and posaconazole otic treatments as well as miconazole shampoo. The clinical histories supported the potential for azole antifungal exposure to lead to reduced miconazole susceptibility.

### 3.3 Molecular Modelling of the *M. pachydermatis* Lanosterol 14α‐Demethylase Interaction With Miconazole

Molecular modelling was pursued to locate the amino acid substitutions identified in Table 3 within the structure of lanosterol 14α‐demethylase and gauge their potential effect on miconazole binding. A model of *M. pachydermatis* lanosterol 14α‐demethylase complexed with fluconazole was generated using wild‐type (WT) lanosterol 14α‐demethylase sequence predicted from the genome assembly (ATCC 14522) [7]. Residue A302 was located on the I helix deep within the lanosterol 14α‐demethylase azole‐binding pocket and the A302T/A302V mutations were the most obvious to affect miconazole binding.

**FIGURE 2:**
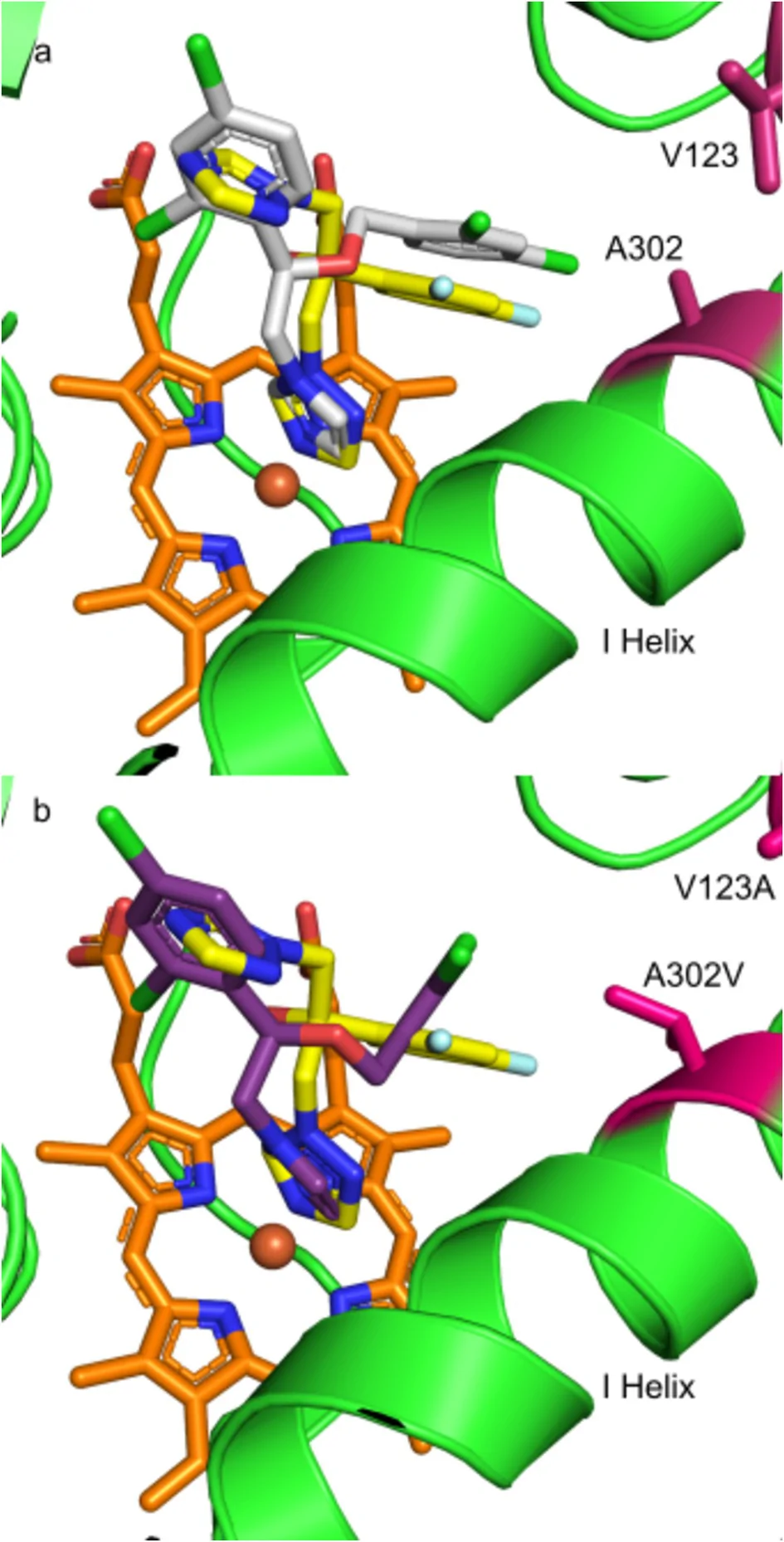
Docking of *R*‐miconazole to the *Malassezia pachydermatis* lanosterol 14α‐demethylase homology model. (a) Docking of *R*‐miconazole (white carbons) to wild‐type lanosterol 14α‐demethylase with residues A302 and V123 (magenta). *R*‐miconazole binds in a similar fashion to fluconazole (yellow carbons) particularly in the parallel orientation of the 2,4‐dihalogenated rings. (b) *R*‐miconazole docked into the A302V V123A mutant lanosterol 14α‐demethylase showing a deviation of binding from that expected. Lanosterol 14α‐demethylase is shown as a green ribbon. Fluconazole, derived from the original crystal structure (PDB ID: 4WMZ) is shown as a reference. The highest‐ranked pose is shown in both cases. Oxygen—red; nitrogen—blue; chlorine—green; fluorine—light blue; iron (Fe) and heme—orange sphere and sticks.

In order to assess the effect of the A302T/A302V mutations on the space in the binding pocket, *R*‐miconazole, the more active miconazole enantiomer [23], was docked into both the WT and A302V mutant proteins. Docking to the WT enzyme revealed that the 2,4‐dichlorobenzoyl group (Figure 2) bound deep into a hydrophobic pocket close to A302. This orientation was parallel to the 2,4‐difluophenyl group of fluconazole from the crystal structure of *S. cerevisiae* lanosterol 14α‐demethylase (where the corresponding residue to A302 is G310) [17]. A cluster of similar poses (< 1.5 Å root mean square deviation) was seen overall for the WT docking. By contrast, docking results using the A302V mutant enzyme showed a significant deviation from the plane of the ring seen in fluconazole as a direct result of the increased size of the amino acid. Numerous poses, without major clusters, supported the conclusion that the larger amino acid (Val—3 carbons) reduced space in the binding pocket relative to the WT Ala (1 carbon; Figure 2). This larger side chain caused steric crowding with ligands binding in this region, particularly the 4‐chloro substituent of *R*‐miconazole. V123 was also located near the miconazole‐binding site.

Additional models were constructed to assess the location and potential influence of other amino acid substitutions on ligand binding. The other amino acid positions were located elsewhere in the lanosterol 14α‐demethylase molecule with only the potential for a remote effect on the ligand‐binding pocket (Figure S1). It was not obvious how these mutations would affect miconazole binding.

## 4 Discussion

Analysis of *M. pachydermatis* from OE cases demonstrated that strains with reduced miconazole susceptibility were found among dogs seen in the routine of a tertiary service. This result supports the idea that resistance can emerge with clinical use of antifungal drugs in dermatological practice. Results presented here, for the first time, placed data regarding *M. pachydermatis* lanosterol 14α‐demethylase amino acid substitutions into the context of the *M. pachydermatis* phylogenetic tree, and provide clarity regarding which substitutions have a demonstrable effect on miconazole susceptibility and which are merely clade‐associated. Previous authors have drawn phylogenetic trees using some of the same loci considered here (ITS, LSU) as well as the chitin synthase 2 gene (*CHS2*) [24, 25, 26, 27, 28]. The fact that 45 of 997 positions at the *ACT1* locus differed among the isolates provided greater ability to discriminate between them compared to loci used in other studies. The resulting tree showed three major clades (Figure 1). This three‐clade structure was further supported by the clade‐specific *ERG11* sequences that were derived to better understand lanosterol 14α‐demethylase–miconazole interactions. Fungal phylogenies are now reported using whole‐genome data that concatenate sequences from dozens of loci to reveal the evolutionary relationship between isolates of the same species [29, 30]. We plan to pursue such analysis for the current collection of OE isolates.

One major problem underlying this area of study, however, is the lack of a standardised assay for measuring *M. pachydermatis* antifungal susceptibility (reviewed by Peano et al. [19]). The CLSI guidelines for broth microdilution assays involve growing yeasts in RPMI 1640 tissue culture medium which does not support *M. pachydermatis* growth [31]. Use of alternative growth media may result in interference with antifungal drug activity [19]. For this reason, we elected to use the disk diffusion assay similar to the CLSI reference assay [15] that was validated for testing *M. pachydermatis* miconazole susceptibility [14]. Although there is not a breakpoint available for the data, the collection of canine OE isolates spanned a wide range of zone‐of‐inhibition diameters in response to a commercial miconazole disk. Zones of inhibition greater than the *M. pachydermatis* type strain indicate susceptibility to the drug while isolates that grew right up to the edge of the disk (9 mm diameter; Table 2) were clearly no longer susceptible to miconazole. Literature‐documented mechanisms that explain *M. pachydermatis* reduced azole susceptibility include *ERG11* mutations that result in lanosterol 14α‐demethylase amino acid substitutions [6, 20, 21, 32, 33] and overproduction of lanosterol 14α‐demethylase from a quadruplicated *ERG11* locus [34]. The action of efflux pumps also has been implicated as a mechanism that contributes to *M. pachydermatis* azole resistance [35].

Among the *M. pachydermatis* isolates characterised here, lanosterol 14α‐demethylase amino acid A302 was the main driver of reduced miconazole susceptibility (Table 3). The association between A302 substitutions and *M. pachydermatis* azole susceptibility was noted by Kano et al. [6] who characterised a canine dermatitis isolate (A302V, M138V) that was described as resistant to both itraconazole and ketoconazole. Kano et al. [21] investigated additional *M. pachydermatis* clinical isolates from dogs with *Malassezia* dermatitis and identified a second multidrug‐resistant strain with the A302V and M138V substitutions. These findings led to development of a PCR‐based molecular assay to detect nucleotide changes associated with A302 substitutions that could facilitate antifungal selection for dogs with *M. pachydermatis* dermatitis or otitis [36]. When interpreted in the context of our current findings, it is likely that these *M. pachydermatis* isolates were from Clade I because they contained few amino acid substitutions compared to the type strain. Studies that examine larger isolate collections and associate antifungal resistance with *ERG11* mutations have generated longer lists of lanosterol 14α‐demethylase amino acid substitutions [32, 33, 37]. Work presented here demonstrates that most of these amino acid sequence changes are associated with clade, rather than affecting interaction between the azole and lanosterol 14α‐demethylase target.

Analysis of lanosterol 14α‐demethylase (CYP51, Erg11) in other fungal species provides additional insight into the interaction between the lanosterol 14α‐demethylase protein and azoles in the context of species for which the CLSI standardised susceptibility tests can be used. The location of A302 in *M. pachydermatis* lanosterol 14α‐demethylase corresponds to amino acid T289 in *Aspergillus fumigatus* CYP51A protein [38] and is part of a mutation that is well known for its resistance to voriconazole with T289 located close to the voriconazole 2,4‐difluoro phenyl moiety [39]. Bédard et al. [40] noted that the LTTPVFG region of lanosterol 14α‐demethylase is highly conserved across the fungal kingdom; it is located from amino acids 119 to 125 in *M. pachydermatis*. Engineering an Ala‐to‐Val change at this location in *Candida albicans* (V125A) led to decreased susceptibility to multiple azole antifungals. Williamson et al. [41] found the V125A substitution in conjunction with F126L in multiple clinical isolates of *Candida auris* that had decreased susceptibility to multiple azoles. In the current study, the isolates from Dog 11 had V123A substitutions. Tools for molecular manipulation of *Malassezia* species are becoming available and set the stage for targeted mutagenesis of *ERG11* to test these ideas [42].

It remains to be determined whether amino acid substitutions in the current work decrease susceptibility to multiple azoles. The lack of a standardised testing assay for *M. pachydermatis* makes comparisons between studies difficult. Diáz et al. [32] demonstrated this point by showing contradictory conclusions regarding azole susceptibility for the same isolate using a disk diffusion assay on MHA compared to SDA. Their overall study used MHA as recommended by Pasquetti et al. [14] which matched the conditions in the current study. Diaz et al. [32] demonstrated decreased fluconazole susceptibility for an isolate that contained the A302T and G459D substitutions, supporting the idea that these changes affect the action of multiple azoles. However, susceptibility of the isolate to ketoconazole was unchanged, suggesting that the substitutions do not confer pan‐azole resistance. Amino acids 459, 460 and 461 were implicated in azole susceptibility in multiple studies [20, 32, 33]. These amino acids are part of an external loop of the lanosterol 14α‐demethylase structure without a clear indication of how they might affect azole binding (Figure S1).

Figure S2 shows the structure of azoles that are indicated for treatment of *M. pachydermatis* in dogs. The structure of miconazole is small (compared to posaconazole), contacting amino acid A302 with V123 in close proximity in the lanosterol 14α‐demethylase drug binding site (Figure 2). Lanosterol 14α‐demethylase structural changes resulting from A302T or A302V mutation would not permit optimal binding of miconazole, leading to decreased susceptibility. Longer‐tailed azole drugs such as itraconazole or posaconazole would have a greater number of contacts within the lanosterol 14α‐demethylase binding cavity, thereby exhibiting a higher binding affinity and antifungal effects even when miconazole is no longer effective (Figure S3). Although further investigation is needed to explore these ideas, the line of reasoning is consistent with local clinical experience that suggests that posaconazole can still be effective in dogs that are no longer responding to miconazole. Such observations would advocate for initial use of miconazole for *M. pachydermatis* OE treatment, reserving the longer‐tailed azoles (e.g., itraconazole, posaconazole) for cases with miconazole treatment failure. Additional attention also is needed to explore the potential emergence of resistance to the longer‐tailed azoles, a situation that would critically limit the ability to treat *M. pachydermatis* OE with topical antifungals.

## 5 Conclusions

Miconazole‐resistant *M. pachydermatis* isolates were among yeasts collected from dogs with a clinical OE diagnosis. Phylogenetic analysis based on four loci grouped the isolates into three clades. DNA sequencing of the *ERG11* gene in each isolate revealed amino acids associated with miconazole resistance as well as sequence differences associated with clades. Molecular modelling demonstrated that amino acid 302 is located within the miconazole‐binding region of lanosterol 14α‐demethylase. *ERG11* mutations that lead to A302 substitutions are associated with decreased miconazole susceptibility. Targeted *ERG11* mutagenesis and standardised antifungal susceptibility assays are needed to test hypotheses regarding combinations of specific lanosterol 14α‐demethylase substitutions and their effects on azole resistance. Effective antifungal stewardship is essential to decrease the development of pan‐azole resistance that would limit treatment options for dogs with *M. pachydermatis*‐associated disease.

## Author Contributions

**Cole M. Belcher:** conceptualisation, methodology, investigation, writing – original draft, writing – review and editing and visualisation. **Clarissa P. Souza:** conceptualisation, methodology, investigation, writing – review and editing, project administration and funding acquisition. **Elyas McGuire:** formal analysis, investigation, writing – review and editing and visualisation. **Chien‐Che Hung:** conceptualisation, methodology, investigation, writing – review and editing, project administration and funding acquisition. **Joel D. A. Tyndall:** conceptualisation, methodology, formal analysis, investigation, writing – review and editing, visualisation, supervision and project administration. **Lois L. Hoyer:** conceptualisation, methodology, investigation, writing – original draft, writing – review and editing, supervision, project administration and funding acquisition.

## Funding

This work was supported by the James R. Harkness Fund, University of Illinois College of Veterinary Medicine and by a grant from the American College of Veterinary Dermatology Research Foundation.

## Conflicts of Interest

The authors declare no conflicts of interest.

## Supporting information


**TABLE S1:** Oligonucleotide primers used for PCR amplification and Sanger DNA sequencing of *Malassezia pachydermatis* loci for phylogenetic analysis.
**TABLE S2:** Oligonucleotide primers used for PCR amplification and Sanger DNA sequencing of the *Malassezia pachydermatis ERG11* gene.
**TABLE S3:** GenBank accession numbers for DNA sequences derived from this study.
**FIGURE S1:** Locations of various amino acids in *Malassezia pachydermatis* lanosterol 14α‐demethylase structure. The figures show the locations of amino acids implicated in interactions with azole antifungals in the context of the current study and in other published literature. The lower figure shows the molecule rotated 90° relative to the top image.
**FIGURE S2:** Chemical structures of azole antifungals that are indicated for treatment of *Malassezia pachydermatis* in dogs. The short‐tailed azoles include miconazole, clotrimazole and fluconazole. A medium‐tailed azole (ketoconazole) and the long‐tailed azoles (posaconazole and itraconazole) also are shown.
**FIGURE S3:** Interaction of posaconazole with *Malassezia pachydermatis* lanosterol 14α‐demethylase. The *M. pachydermatis* lanosterol 14α‐demethylase model is shown as green ribbon with heme as orange sticks. Posaconazole (purple sticks) was derived from the superimposition of the *Saccharomyces cerevisiae* lanosterol 14α‐demethylase (CYP51) – posaconazole complex (PDB ID: 6E8Q). Molecule weight of fluconazole (306.3 Da) and posaconazole (700.8 Da) for comparison.

## Data Availability

All data are either reported in this manuscript or were deposited into public databases such as GenBank except the lanosterol 14α‐demethylase coordinates that are available upon request.

## References

[1] G. Zur , B. Lifshitz , and T. Bdolah‐Abram , “The Association Between the Signalment, Common Causes of Canine Otitis Externa and Pathogens,” Journal of Small Animal Practice 52 (2011): 254–258. 21539570 10.1111/j.1748-5827.2011.01058.x

[2] D. G. O'Neill , D. B. Church , P. D. McGreevy , P. C. Thomson , and D. C. Brodbelt , “Prevalence of Disorders Recorded in Dogs Attending Primary‐Care Veterinary Practices in England,” PLoS One 9 (2014): e90501. 24594665 10.1371/journal.pone.0090501 PMC3942437

[3] L. R. Perry , B. MacLennan , R. Korven , and T. A. Rawlings , “Epidemiological Study of Dogs With Otitis Externa in Cape Breton, Nova Scotia,” Canadian Veterinary Journal 58 (2017): 168–174. PMC5234316 28216686

[4] J. M. Boone , R. Bond , A. Loeffler , E. A. Ferguson , and A. Hendricks , “ *Malassezia* Otitis Unresponsive to Primary Care: Outcome in 59 Dogs,” Veterinary Dermatology 32 (2021): 441. 34189776 10.1111/vde.12995

[5] T. Nuttall and E. Bensignor , “A Pilot Study to Develop an Objective Clinical Score for Canine Otitis Externa,” Veterinary Dermatology 25 (2014): 530–537. 25130194 10.1111/vde.12163

[6] R. Kano , S. Yokoi , N. Kariya , K. Oshimo , and H. Kamata , “Multi‐Azole‐Resistant Strain of *Malassezia pachydermatis* Isolated From a Canine *Malassezia* Dermatitis,” Medical Mycology 57 (2019): 346–350. 29800467 10.1093/mmy/myy035

[7] L. L. Hoyer , C. P. Souza , C.‐C. Hung , E. K. Hogan , and A. G. Hernandez , “A Telomere‐To‐Telomere Genome Assembly for *Malassezia pachydermatis* ATCC 14522 (CBS 1879),” Microbiology Resource Announcements 14 (2025): e0012525. 40552812 10.1128/mra.00125-25 PMC12352072

[8] F. Madeira , N. Madhusoodanan , J. Lee , et al., “The EMBL‐EBI Job Dispatcher Sequence Analysis Tools Framework in 2024,” Nucleic Acids Research 52, no. W1 (2024): 521–525. 10.1093/nar/gkae241 PMC11223882 38597606

[9] P. Stothard , “The Sequence Manipulation Suite: JavaScript Programs for Analyzing and Formatting Protein and DNA Sequences,” BioTechniques 28 (2000): 1102–1104. 10868275 10.2144/00286ir01

[10] K. Katoh and D. M. Standley , “MAFFT Multiple Sequence Alignment Software Version 7: Improvements in Performance and Usability,” Molecular Biology and Evolution 30 (2013): 772–780. 23329690 10.1093/molbev/mst010 PMC3603318

[11] D. Darriba , D. Posada , A. M. Kozlov , A. Stamatakis , B. Morel , and T. Flouri , “ModelTest‐NG: A New and Scalable Tool for the Selection of DNA and Protein Evolutionary Models,” Molecular Biology and Evolution 37 (2020): 291–294. 31432070 10.1093/molbev/msz189 PMC6984357

[12] L.‐T. Nguyen , H. A. Schmidt , A. von Haeseler , and B. Q. Minh , “IQ‐TREE: A Fast and Effective Stochastic Algorithm for Estimating Maximum‐Likelihood Phylogenies,” Molecular Biology and Evolution 32 (2015): 268–274. 25371430 10.1093/molbev/msu300 PMC4271533

[13] I. Letunic and P. Bork , “Interactive Tree of Life (iTOL) v6: Recent Updates to the Phylogenetic Tree Display and Annotation Tool,” Nucleic Acids Research 52, no. W1 (2024): 78–82. 10.1093/nar/gkae268 PMC11223838 38613393

[14] M. Pasquetti , E. Chiavassa , P. Tizzani , P. Danesi , and A. Peano , “Agar Diffusion Procedures for Susceptibility Testing of *Malassezia pachydermatis*: Evaluation of Mueller‐Hinton Agar Plus 2% Glucose and 0.5 μg/ml Methylene Blue as the Test Medium,” Mycopathologia 180 (2015): 153–158. 26138434 10.1007/s11046-015-9913-2

[15] CLSI , “Method for Antifungal Disk Diffusion Susceptibility Testing of Yeasts; Approved Guideline,” in CLSI Document M44‐A2, 2nd ed. (Clinical and Laboratory Standards Institute, 2009).

[16] B. Webb and A. Sali , “Comparative Protein Structure Modeling Using MODELLER,” Current Protocols in Bioinformatics 54 (2016): 5.6.1–5.6.37. 10.1002/cpbi.3 PMC5031415 27322406

[17] A. A. Sagatova , M. V. Keniya , R. K. Wilson , B. C. Monk , and J. D. A. Tyndall , “Structural Insights Into Binding of the Antifungal Drug Fluconazole to *Saccharomyces cerevisiae* Lanosterol 14α‐Demethylase,” Antimicrobial Agents and Chemotherapy 59 (2015): 4982–4989. 26055382 10.1128/AAC.00925-15 PMC4505223

[18] G. Jones , P. Willett , R. C. Glen , A. R. Leach , and R. Taylor , “Development and Validation of a Genetic Algorithm for Flexible Docking,” Journal of Molecular Biology 267 (1997): 727–748. 9126849 10.1006/jmbi.1996.0897

[19] A. Peano , M. Pasquetti , P. Tizzani , E. Chiavassa , J. Guillot , and E. Johnson , “Methodological Issues in Antifungal Susceptibility Testing of *Malassezia pachydermatis* ,” Journal of Fungi 3 (2017): 37. 29371554 10.3390/jof3030037 PMC5715951

[20] R. Kano and H. Kamata , “Miconazole‐Tolerant Strains of *Malassezia pachydermatis* Generated by Culture in Medium Containing Miconazole,” Veterinary Dermatology 31 (2020): 97–101. 31729813 10.1111/vde.12805

[21] R. Kano , C. Aramaki , N. Murayama , et al., “High Multi‐Azole‐Resistant *Malassezia pachydermatis* Clinical Isolates From Canine *Malassezia* Dermatitis,” Medical Mycology 58 (2020): 197–200. 31329927 10.1093/mmy/myz037

[22] M. V. Keniya , M. Sabherwal , R. K. Wilson , et al., “Crystal Structures of Full‐Length Lanosterol 14α‐Demethylases of Prominent Fungal Pathogens *Candida albicans* and *Candida glabrata* Provide Tools for Antifungal Discovery,” Antimicrobial Agents and Chemotherapy 62 (2018): e01134‐18. 30126961 10.1128/AAC.01134-18 PMC6201081

[23] Y. Du , L. Luo , S. Sun , Z. Jiang , and X. Guo , “Enantioselective Separation and Determination of Miconazole in Rat Plasma by Chiral LC–MS/MS: Application in a Stereoselective Pharmacokinetic Study,” Analytical and Bioanalytical Chemistry 409 (2017): 6315–6323. 28852798 10.1007/s00216-017-0551-z

[24] C. Cafarchia , M. S. Latrofa , G. Testini , et al., “Molecular Characterization of *Malassezia* Isolates From Dogs Using Three Distinct Genetic Markers in Nuclear DNA,” Molecular and Cellular Probes 21 (2007): 229–238. 17320347 10.1016/j.mcp.2007.01.002

[25] S.‐H. Han , T.‐H. Chung , E.‐H. Nam , S.‐H. Park , and C.‐Y. Hwang , “Molecular Analysis of *Malassezia pachydermatis* Isolated From Canine Skin and Ear in Korea,” Medical Mycology 51 (2013): 396–404. 23167706 10.3109/13693786.2012.740575

[26] L. Puig , G. Castellá , and F. J. Cabañes , “Cryptic Diversity of *Malassezia pachydermatis* From Healthy and Diseased Domestic Animals,” Mycopathologia 181 (2016): 681–688. 27283291 10.1007/s11046-016-0026-3

[27] U. Czyzewska , M. Bartoszewicz , M. Siemieniuk , and A. Tylicki , “Genetic Relationships and Population Structure of *Malassezia pachydermatis* Strains Isolated From Dogs With Otitis Externa and Healthy Dogs,” Mycologia 110 (2018): 666–676. 30130476 10.1080/00275514.2018.1495981

[28] S. Hađina , B. Bruvo Mađarić , S. Kazazić , et al., “ *Malassezia pachydermatis* From Brown Bear: A Comprehensive Analysis Reveals Novel Genotypes and Distribution of All Detected Variants in Domestic and Wild Animals,” Frontiers in Microbiology 14 (2023): 1151107. 37275156 10.3389/fmicb.2023.1151107 PMC10236562

[29] J. T. Ladner , J. M. Palmer , C. L. Ettinger , et al., “The Population Genetics of the Causative Agent of Snake Fungal Disease Indicate Recent Introductions to the USA,” PLoS Biology 20 (2022): e3001676. 35737674 10.1371/journal.pbio.3001676 PMC9223401

[30] A. Menicucci , S. Iacono , M. Ramos , et al., “Can Whole Genome Sequencing Resolve Taxonomic Ambiguities in Fungi? The Case Study of *Colletotrichum* Associated With Ferns,” Frontiers in Fungal Biology 6 (2025): 1540469. 40093768 10.3389/ffunb.2025.1540469 PMC11906685

[31] CLSI , ed., “Reference Method for Broth Dilution Antifungal Susceptibility Testing of Yeasts – 4th Ed,” in CLSI Document M27 (Clinical and Laboratory Standards Institute, 2017).

[32] L. Díaz , G. Castellá , M. R. Bragulat , and F. J. Cabañes , “ *ERG11* Gene Variability and Azole Susceptibility in *Malassezia pachydermatis* ,” Mycopathologia 188 (2023): 21–34. 36495417 10.1007/s11046-022-00696-9 PMC10169892

[33] S. Álvarez‐Pérez , S. Quevedo‐Caraballo , M. E. García , and J. L. Blanco , “Prevalence and Genetic Diversity of Azole‐Resistant *Malassezia pachydermatis* Isolates From Canine Otitis and Dermatitis: A 2‐Year Study,” Medical Mycology 62 (2024): myae053. 38734886 10.1093/mmy/myae053

[34] M. Kim , Y.‐J. Cho , M. Park , Y. Choi , S. Y. Hwang , and W. H. Jung , “Genomic Tandem Quadruplication is Associated With Ketoconazole Resistance in *Malassezia pachydermatis* ,” Journal of Microbiology and Biotechnology 28 (2018): 1937–1945. 30562885 10.4014/jmb.1810.10019

[35] R. Iatta , M. R. Puttili , D. Immediato , D. Otranto , and C. Cafarchia , “The Role of Drug Efflux Pumps in *Malassezia pachydermatis* and *Malassezia furfur* Defence Against Azoles,” Mycoses 60 (2017): 178–182. 27774659 10.1111/myc.12577

[36] R. Kano and N. Murayama , “Rapid Molecular Detection of Antifungal‐Resistant Strains of *Malassezia pachydermatis* ,” Medical Mycology Journal 63 (2022): 53–56. 35650071 10.3314/mmj.21-00013

[37] M. Domán , D. Első , K. Pintér , E. Wehmann , E. Fehér , and T. Magyar , “Antifungal Susceptibility of *Malassezia pachydermatis* Isolates From Companion Animals and Genomic Insights Into Resistance Mechanisms,” Antibiotics 14 (2025): 902. 41009881 10.3390/antibiotics14090902 PMC12466836

[38] E. Snelders , S. M. T. Camps , A. Karawajczyk , et al., “Genotype‐Phenotype Complexity of the TR_46_/Y121F/T289A *cyp*51*A* Azole Resistance Mechanism in *Aspergillus fumigatus* ,” Fungal Genetics and Biology 82 (2015): 129–135. 26092193 10.1016/j.fgb.2015.06.001

[39] P. Hosseini , M. V. Keniya , A. A. Sagatova , et al., “The Molecular Basis of the Intrinsic and Acquired Resistance to Azole Antifungals in *Aspergillus fumigatus* ,” Journal of Fungi 10 (2024): 820. 39728316 10.3390/jof10120820 PMC11678245

[40] C. Bédard , I. Gagnon‐Arsenault , J. Boisvert , et al., “Most Azole Resistance Mutations in the *Candida albicans* Drug Target Confer Cross‐Resistance Without Intrinsic Fitness Cost,” Nature Microbiology 9 (2024): 3025–3040. 10.1038/s41564-024-01819-2 39379635

[41] B. Williamson , A. Wilk , K. D. Guerrero , et al., “Impact of Erg11 Amino Acid Substitutions Identified in *Candida* *auris* Clade III Isolates on Triazole Drug Susceptibility,” Antimicrobial Agents and Chemotherapy 66 (2022): e01624‐21. 34633842 10.1128/AAC.01624-21 PMC8765314

[42] G. Ianiri and J. Heitman , “Approaches for Genetic Discoveries in the Skin Commensal and Pathogenic *Malassezia* Yeasts,” Frontiers in Cellular and Infection Microbiology 10 (2020): 393. 32850491 10.3389/fcimb.2020.00393 PMC7426719

