# Supplementary material for: Phylogenetic and Structural Analysis of Miconazole Susceptibility in Malassezia pachydermatis Isolates From Dogs With Otitis Externa
Source: Vet Dermatol. 2026 Mar 19;37(5):620–9. doi: 10.1111/vde.70059 (PMC13569138; doi:10.1111/vde.70059)
Supplement: Supplementary file 1 [file VDE-37-620-s001.docx]

**TABLE S1** | Oligonucleotide primers used for PCR amplification and Sanger DNA sequencing of *Malassezia pachydermatis* loci for phylogenetic analysis.

Locus	Primer	Sequence (5’ – 3’)

ITS	ITS4	TCC TCC GCT TAT TGA TAT GC

ITS5	GGA AGT AAA AGT CGT AAC AAG G

18S rDNA (SSU)	NS3	GCA AGT CTG GTG CCA GCA GCC

NS6	GCA TCA CAG ACC TGT TAT TGC CTC

26S rDNA (LSU)	NL-1	GCA TAT CAA TAA GCG GAG GAA AAG

NL-4	GGT CCG TGT TTC AAG ACG G

*ACT1*	MpACT1F	CGC CGA AAT GTT TCT TCC CTT C

MpACT1R	TAC CTG GGT CCT CTT GGT ATG

The ITS and 18S rDNA (SSU) primers were from White TJ, Bruns T, Lee S, Taylor J. Amplification and direct sequencing of fungal ribosomal RNA genes for phylogenetics. In: PCR Protocols: A Guide to Methods and Applications. Academic Press, San Diego. 1990;315-22.

The 26S (LSU) primers were from O’Donnell K. *Fusarium* and its near relatives. In: The Fungal Holomorph: Mitotic, Meiotic and Pleomorphic Speciation in Fungal Systematics. CAB International, Wallingford, UK. 225-33.

The *ACT1* primers were designed from the whole genome sequence for *M. pachydermatis* strain ATCC 14522 (ASM4736803v1).

Hoyer LL, Souza CP, Hung C-C, Hogan EK, Hernandez AG. A telomere-to-telomere genome assembly for *Malassezia pachydermatis* ATCC 14522 (CBS 1879). Microbiol Resour Announc. 2025;14:e0012525.

**TABLE S2** | Oligonucleotide primers used for PCR amplification and Sanger DNA sequencing of the *Malassezia pachydermatis ERG11* gene.

Primer	Orientation	Coordinates	Sequence (5’ – 3’)

MpERG11F	F	-310 to -286	CTC TCC ACT CGC TTC AGT CAC ATT T

MpERG11R	R	+88 to +64	GAA CCA CAT CGC GTT TCG CTA TAC T

MALAERG1S	F	1 to 20	ATG TCA GAC TTG ATT TCT AT

MALAERG2S	F	431 to 450	TCA AGT CAG GTC TCT CGA CG

MALAERG3S	F	811 to 830	GGT ACG GAC CAC GAT ATG AT

MALAERG4S	F	1331 to 1350	AGG CGA AGG GTC TTG AGC TG

MALAERG1R	R	565 to 546	AAA TGT CCA CCG TCA CAG AC

MALAERG2R	R	950 to 931	CAG CTA CCA GTA GCA CTG GA

MALAERG3R	R	1404 to 1385	GTT ACC GCC AGT GCT GAC C

MALAERG4R	R	1623 to 1604	TTA CTT GGC CAG CGC GGG GC

Primers MpERG11F and MpERG11R were designed from the whole genome sequence for *M. pachydermatis* strain ATCC 14522 (ASM4736803v1).

Hoyer LL, Souza CP, Hung C-C, Hogan EK, Hernandez AG. A telomere-to-telomere genome assembly for *Malassezia pachydermatis* ATCC 14522 (CBS 1879). Microbiol Resour Announc. 2025;14:e0012525.

MALAERG primers were designed from the sequence of a cDNA molecule. The primers began at the start codon (ATG) of *ERG11* and ended at its stop codon (TAA). The sequence length was 1623 bp.

Kano R, Yokoi S, Kariya N, Oshimo K, Kamata H. Multi-azole-resistant strain of *Malassezia pachydermatis* isolated from a canine *Malassezia* dermatitis. Med Mycol. 2019;57:346-50.

**TABLE S3** | GenBank accession numbers for DNA sequences derived from this study.

Isolate	ITS	*ACT1*	18S rDNA	26S rDNA	*ERG11*

ATCC 14522	PV759682	PV764875	PV759646	PV759664	PV764893

11_L	PV759683	PV764876	PV759647	PV759665	PV764894

11_R	PV759684	PV764877	PV759648	PV759666	PV764895

12_L	PV759685	PV764878	PV759649	PV759667	PV764896

12_R	PV759686	PV764879	PV759650	PV759668	PV764897

1_L	PV759687	PV764880	PV759651	PV759669	PV764898

6_L	PV759688	PV764881	PV759652	PV759670	PV764900

4_L	PV759689	PV764882	PV759653	PV759671	PV764901

5_L	PV759690	PV764883	PV759654	PV759672	PV764902

6_R	PV759691	PV764884	PV759655	PV759673	PV764899

8_R	PV759692	PV764885	PV759656	PV759674	PV764904

8_L	PV759693	PV764886	PV759657	PV759675	PV764905

9_R	PV759694	PV764887	PV759658	PV759676	PV764906

10_L	PV759695	PV764888	PV759659	PV759677	PV764907

7_R	PV759697	PV764890	PV759661	PV759679	PV764909

5_R	PV759698	PV764891	PV759662	PV759680	PV764903

2_R	PV759699	PV764892	PV759663	PV759681	PV764910

CBS 10434	NR_126115.1	From Ref Seq^†^	NG_063001.1	NG_066182.1	Not used

^†^*Malassezia caprae* was used as the outgroup for the phylogenetic analysis. The *M. caprae* reference sequence (Ref Seq) is from strain CBS 10434 with GenBank accession number ASM2954288v1 (GCA_029542885.1). The *ACT1* sequence was retrieved from the genome assembly using an *ACT1* BLAST query on the genome webpage.

**FIGURE S1** | Locations of various amino acids in *Malassezia pachydermatis* lanosterol 14α-demethylase structure. The figures show the locations of amino acids implicated in interactions with azole antifungals in the context of the current study and in other published literature. The lower figure shows the molecule rotated 90° relative to the top image.

**FIGURE S2** | Chemical structures of azole antifungals that are indicated for treatment of *Malassezia pachydermatis* in dogs. The short-tailed azoles include miconazole, clotrimazole, and fluconazole. A medium-tailed azole (ketoconazole) and the long-tailed azoles (posaconazole and itraconazole) are also shown.

**FIGURE S3** | Interaction of posaconazole with *Malassezia pachydermatis* lanosterol 14α-demethylase. The *M. pachydermatis* lanosterol 14α-demethylase model is shown as green ribbon with heme as orange sticks. Posaconazole (purple sticks) was derived from the superimposition of the *S. cerevisiae* lanosterol 14α-demethylase (CYP51) – posaconazole complex (PDB ID: 6E8Q) described by Monk et al. (2019). Molecule weight of fluconazole (306.3 Da) and posaconazole (700.8 Da) for comparison.

Monk BC, Keniya MV, Sabherwal M, Wilson RK, Graham DO, Hassan HF, et al. Azole resistance reduces susceptibility to the tetrazole antifungal VT-1161. Antimicrob Agents Chemother 2019;63:10.1128/aac.02114-18.
